# Supplementary material for: Effect of the Administration of a Lyophilised Faecal Capsules on the Intestinal Microbiome of Dogs: A Pilot Study
Source: Genes (Basel). 2023 Aug 25;14(9):1676. doi: 10.3390/genes14091676 (PMC10530739; doi:10.3390/genes14091676)
Supplement: Supplementary file 1 [file genes-14-01676-s001.zip › Supplementary File 1.pdf]

Supplementary File 1

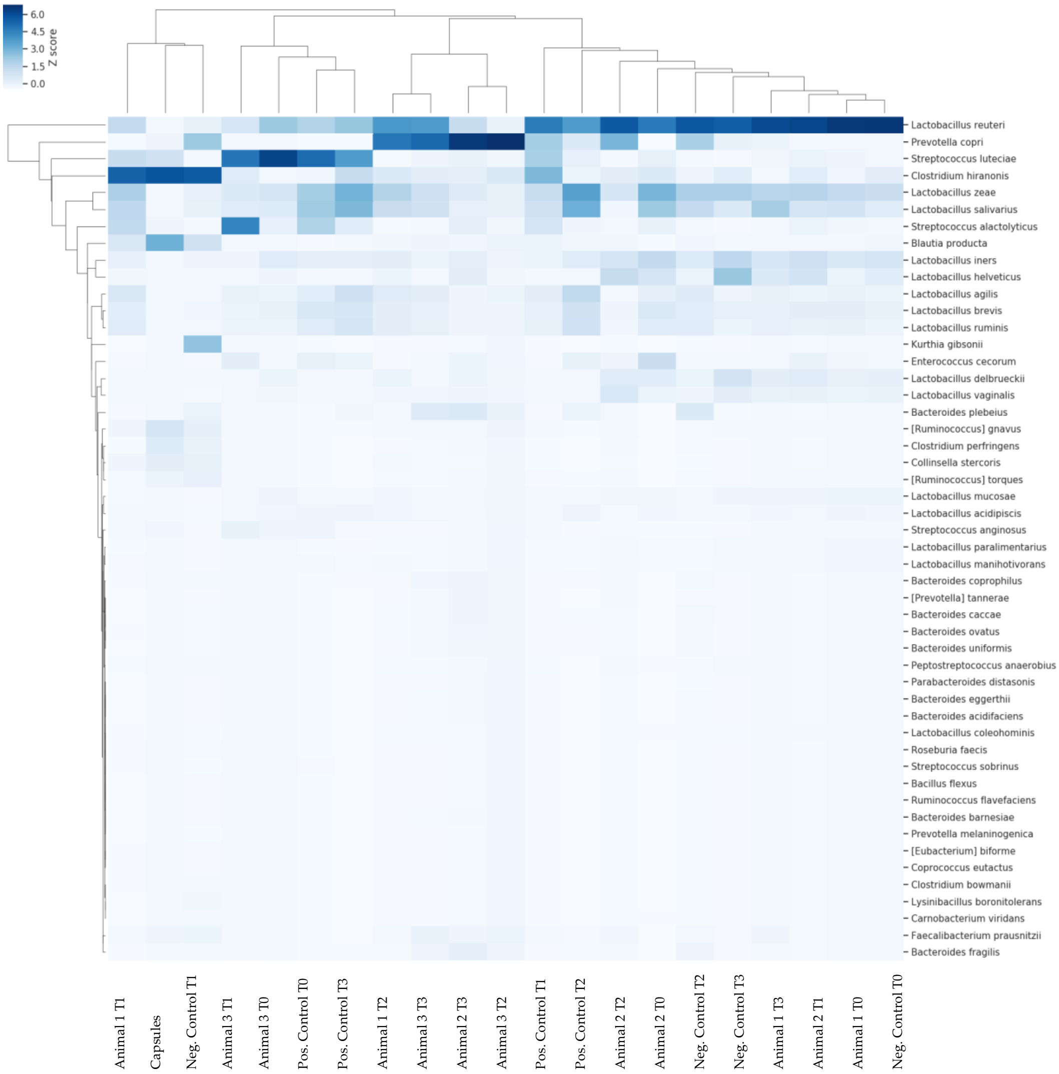

Figure S1. Heatmap representing the 50 OTU with the highest sum of read counts across all samples. The Z value is calculated using the following formula: $\frac{x - \mu \ \text{column}}{\sigma \ \text{column}}$, where μ is the column mean and σ is the column standard deviation. In this case, higher values are represented by darker colours and lower values by lighter colours. Of the phylogenetic trees represented, the one present at the top is noteworthy, since it depicts the grouping of the samples based on the similarity of the composition of the bacterial populations.
