# Supplementary material for: Effect of the Administration of a Lyophilised Faecal Capsules on the Intestinal Microbiome of Dogs: A Pilot Study
Source: Genes (Basel). 2023 Aug 25;14(9):1676. doi: 10.3390/genes14091676 (PMC10530739; doi:10.3390/genes14091676)
Supplement: Supplementary file 1 [file genes-14-01676-s001.zip › Supplementary File 2.pdf]

Supplementary File 2

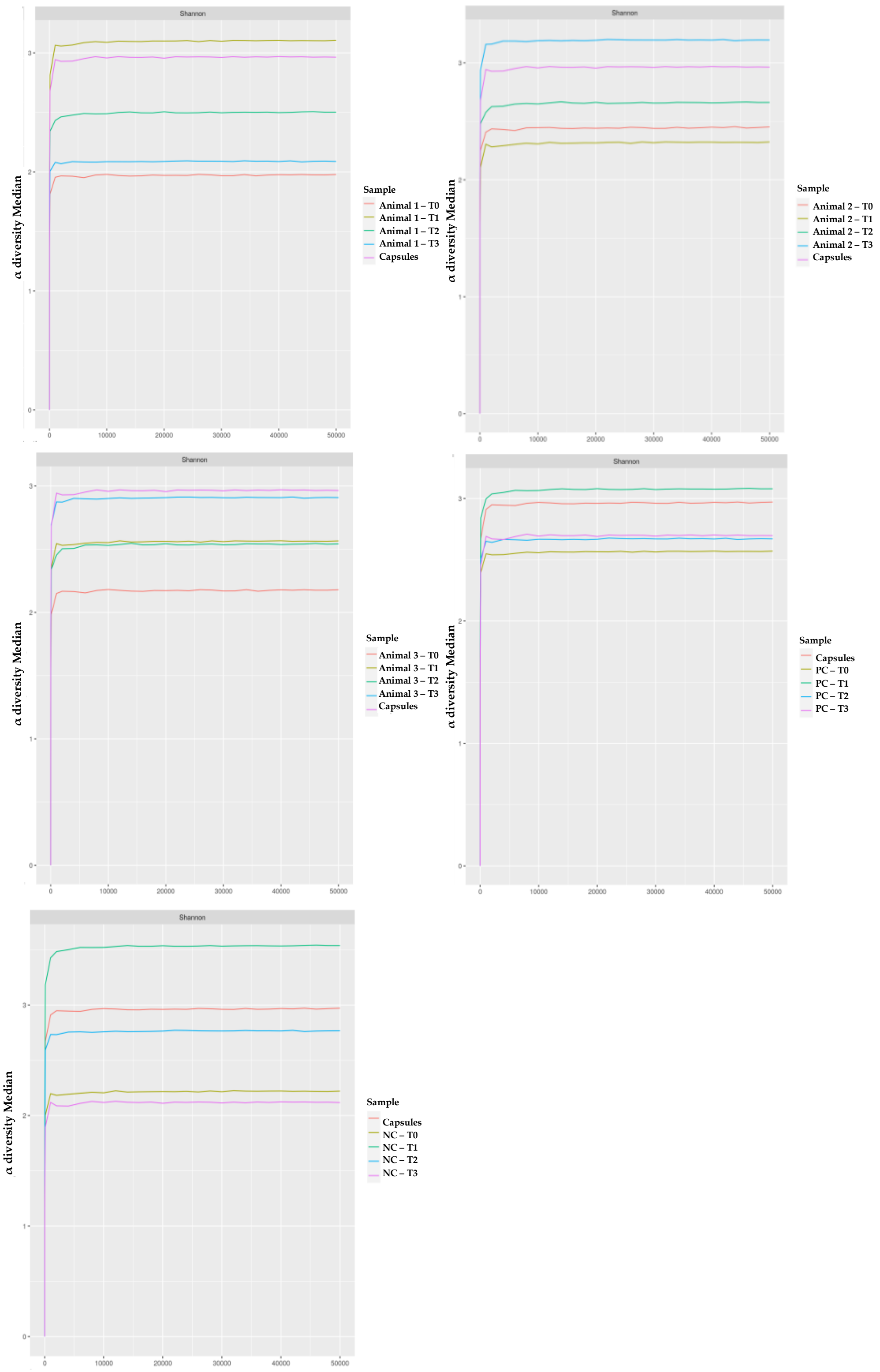

Figure S2. Representation of the α diversity, via Shannon's index, of each faecal sample compared to that present in the FMT capsules.
