# Supplementary material for: Effect of the Administration of a Lyophilised Faecal Capsules on the Intestinal Microbiome of Dogs: A Pilot Study
Source: Genes (Basel). 2023 Aug 25;14(9):1676. doi: 10.3390/genes14091676 (PMC10530739; doi:10.3390/genes14091676)
Supplement: Supplementary file 1 [file genes-14-01676-s001.zip › Supplementary File 3.pdf]

Supplementary File 3

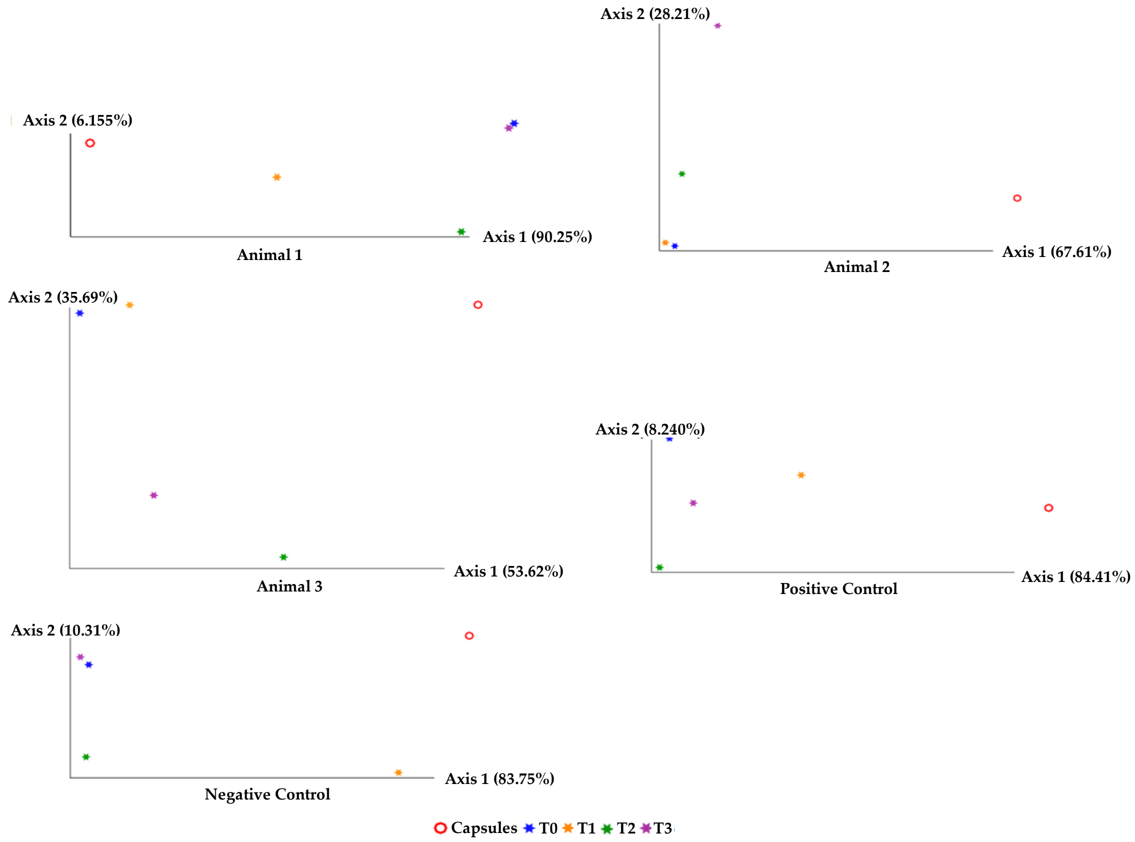

Figure S3. β Diversity expressed through a PCoA plot, depicting the differences present between faecal samples belonging to the same animal.
